# Granular Cell Tumour of the Larynx

**DOI:** 10.1007/s12105-016-0736-3

**Published:** 2016-06-20

**Authors:** Rhona Sproat, Gentle Wong, John Rubin

**Affiliations:** Royal National Throat, Nose and Ear Hospital, 330 Grays Inn Rd, London, WC1X 8DA UK

**Keywords:** Larynx, Granular cell tumour, Histopathology, Surgery

## Abstract

A 55-year-old lady with a 6 month history of hoarse voice presented to our ENT department. Endoscopic examination displayed a white left arytenoid lesion. Biopsy of this lesion displayed a nodule covered with non-keratinizing stratified squamous epithelium, with a central core of polygonal cells, positive for S-100 staining. This confirmed a granular cell tumour. CO_2_ laser was utilised to excise this benign tumour. Granular cell tumours of the head and neck are common, but are infrequently found in the larynx. This case report highlights the importance for the otolaryngologist to be aware of this differential diagnosis, particularly as histologically they may be confused with squamous cell carcinoma.

## Background

Granular cell tumours share several histological features with squamous cell carcinoma. The presence of overlying pseudoepitheliomatous hyperplasia and occasional normal mitosis can provide confusion. Immunohistochemical staining can provide a confirmative diagnosis. Treatment by complete surgical resection is normally successful with low recurrence rates. Failure to recognise this benign lesion from others including squamous cell carcinoma may result in unnecessary treatment.

## Case Presentation

We present the case of a 55 year-old lady who presented with a 6 month history of dysphonia. She complained of deep voice and intermittently losing her voice, particularly at the end of the day. She had no swallowing or breathing difficulties.

Past medical history included acid reflux, arthritis, hypertension and hay fever. Medications included a statin and amlodipine. She has no family history of head and neck cancer. She has smoked three cigarettes a day for many years, and drinks approximately half a bottle of wine at night.

**Fig. 1:**
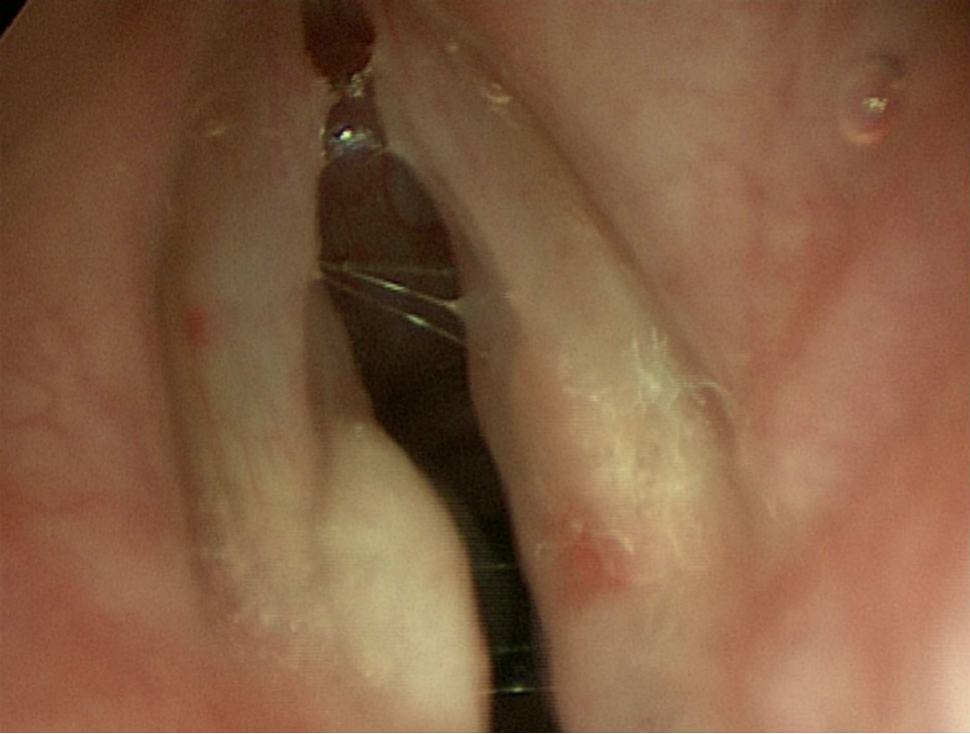
Clinical photograph of endoscopic view of larynx showing left arytenoid lesion

Neck examination was normal. Endoscopic results of the initial examination are displayed (Fig. 1), showing a pale lesion in the left posterior vocal cord. Vocal cord movement, as displayed by video stroboscopy, was symmetrical and normal. Nevertheless, there was cause for concern that this could represent malignancy so we proceeded with further investigations.

## Investigations and Treatment

**Fig. 2:**
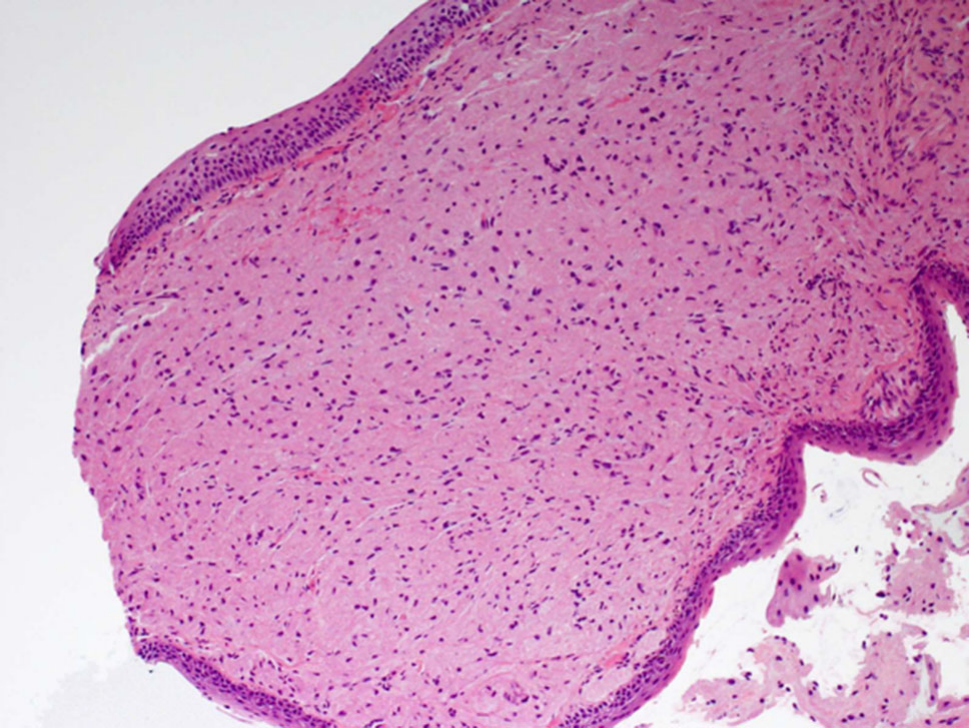
Histology of excised nodule

**Fig. 3:**
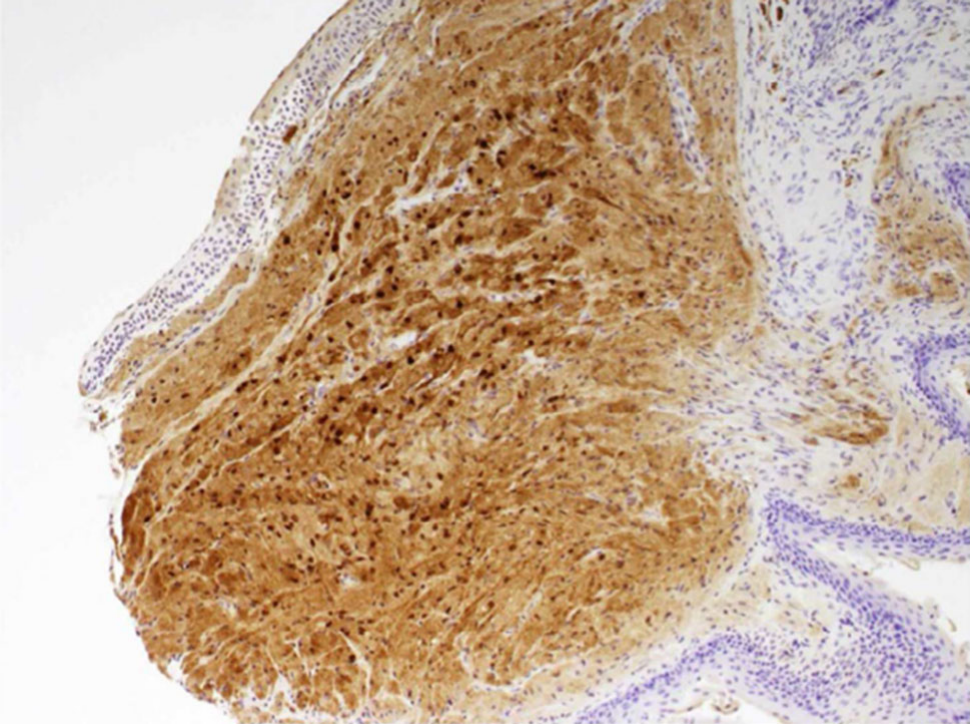
Histology of excised nodule demonstrating positivity to S-100 staining

This lady underwent microlaryngoscopy and biopsy of the left arytenoid lesion under general anaesthetic. Biopsy results displayed a nodule covered by non-keratinizing stratified squamous epithelium with a central core composed of tightly packed large, polygonal cells with abundant granular cytoplasm (Fig. 2). S-100 staining was positive (Fig. 3). A further procedure was performed using CO_2_ laser to remove residual tumour. Histology confirmed a negative free margin.

## Outcome and Follow Up

The patient has completely recovered from the procedures. She continues to be monitored and there remains no evidence of recurrence.

## Discussion

Granular cell tumours, also known as Abrikosoff’s tumours, were first described in 1926. Granular cell tumours are benign neoplasms, thought to be of neuroectodermal origin [1]; and are common in the head and neck region [2], although less than 10 % of such cases are reported in the larynx [3]. They can present in both paediatric and adult populations, and have female preponderance [2].

Granular cell tumours are present in the sub-mucous layer, and appear as a pale mass on the vocal folds (Fig. 1). These have been described both at the glottic level, particularly the posterior glottis, and the subglottis [4, 5]. Resultantly, laryngeal granular cell tumours most commonly present with hoarseness, but can also manifest as dysphagia and globus pharyngeus [4, 6].

Histologically, the presence of overlying pseudoepitheliomatous hyperplasia and occasional normal mitosis can provide confusion with squamous cell carcinoma; however, lack of nuclear hyperplasia or pleomorphisms, and presence of granular cells can differentiate this condition [4]. Granular cell tumours classically have polygonal or elongated cells with eosinophilic granules, small nuclei, and absent mitosis [6]. Positive S100 staining for fibrovascular stroma separating these cells can help to confirm this diagnosis (Fig. 3), as can periodic acid schiff staining for cellular lysosomes present within granular cells [4].

Treatment is by local excision, with recurrence rates at only around 2-3 % if completely excised [7], but there is a 21 % recurrence rate for tumours excised without definitive excision margins [2]. CO_2_ laser excision has proven an adequate means of removing the tumour with negative margins [8]. Malignant transformation occurs in around 1–2 % of granular cell tumours, but only one case has been recorded of malignancy in laryngeal granular cell tumours [9].

In summary, granular cell tumours are an important differential diagnosis for squamous cell carcinoma of the larynx. Where the tumour is completely excised, patients can be reassured of low recurrence rates.

## Learning Points

- Granular cell tumours are a rare cause of a laryngeal mass. It is important to be aware of this histological diagnosis, to avoid confusion with squamous cell carcinoma.
- GCTs are largely benign neoplasms.
- Recurrence is unlikely following complete resection.
- Regular follow-ups are recommended to ensure complete recovery.
